# Supplementary material for: Leukemia inhibitory factor suppresses hepatic de novo lipogenesis and induces cachexia in mice
Source: Nat Commun. 2024 Jan 20;15:627. doi: 10.1038/s41467-024-44924-w (PMC10799847; doi:10.1038/s41467-024-44924-w)
Supplement: Supplementary file 1 — Supplementary Information [file 41467_2024_44924_MOESM1_ESM.pdf]

Supplementary Figures and Tables

Supplementary Figure 1

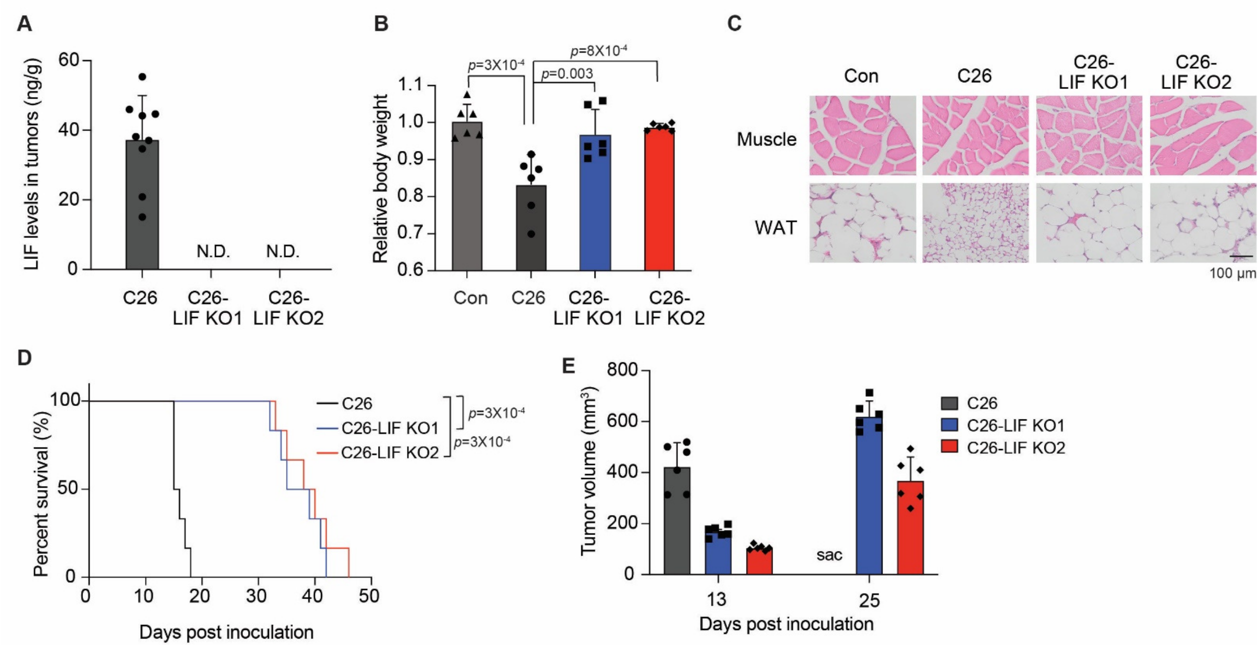

Supplementary Figure 1. LIF produced from C26 tumors contributes to cachexia in C26 tumor-bearing mice.

A. LIF levels in tumors from C26 tumor-bearing mice (n=9) and C26-LIF KO tumor-bearing mice (n=6) determined by ELISA assays. Two different C26-LIF KO cell lines were used for tumorigenesis assays. **B.** The body weight of C26 or C26-LIF KO tumor-bearing mice when the tumors were at the size of ~ 400 mm^3. The body weight of non-tumor bearing Balb/c mice served as control (Con) (n=6/group). **C.** Representative H&E images of muscle and WAT from non-tumor-bearing Balb/c mice and Balb/c mice bearing with C26 or C26-LIF KO tumors when the tumor sizes reached ~ 400 mm^3. At least three independent biological replicates were performed. **D.** Kaplan-Meier survival curves of C26 or C26-LIF KO tumor-bearing mice (n=6/group). The day of tumor cell inoculation was denoted as D0. **E.** The volume of syngeneic xenograft tumors formed by C26 or C26-LIF KO cells (n=6/group). All data are presented as mean ± SD. Both female and male mice were used. sac: mice reached the humane endpoint and were sacrificed before the date of measurement. N.D.: non-detectable. For **B:** One-way ANOVA followed by Tukey's multiple comparison test. For **D**: Kaplan-Meier survival analysis. Source data are provided as Source Data file.

Supplementary Figure 2

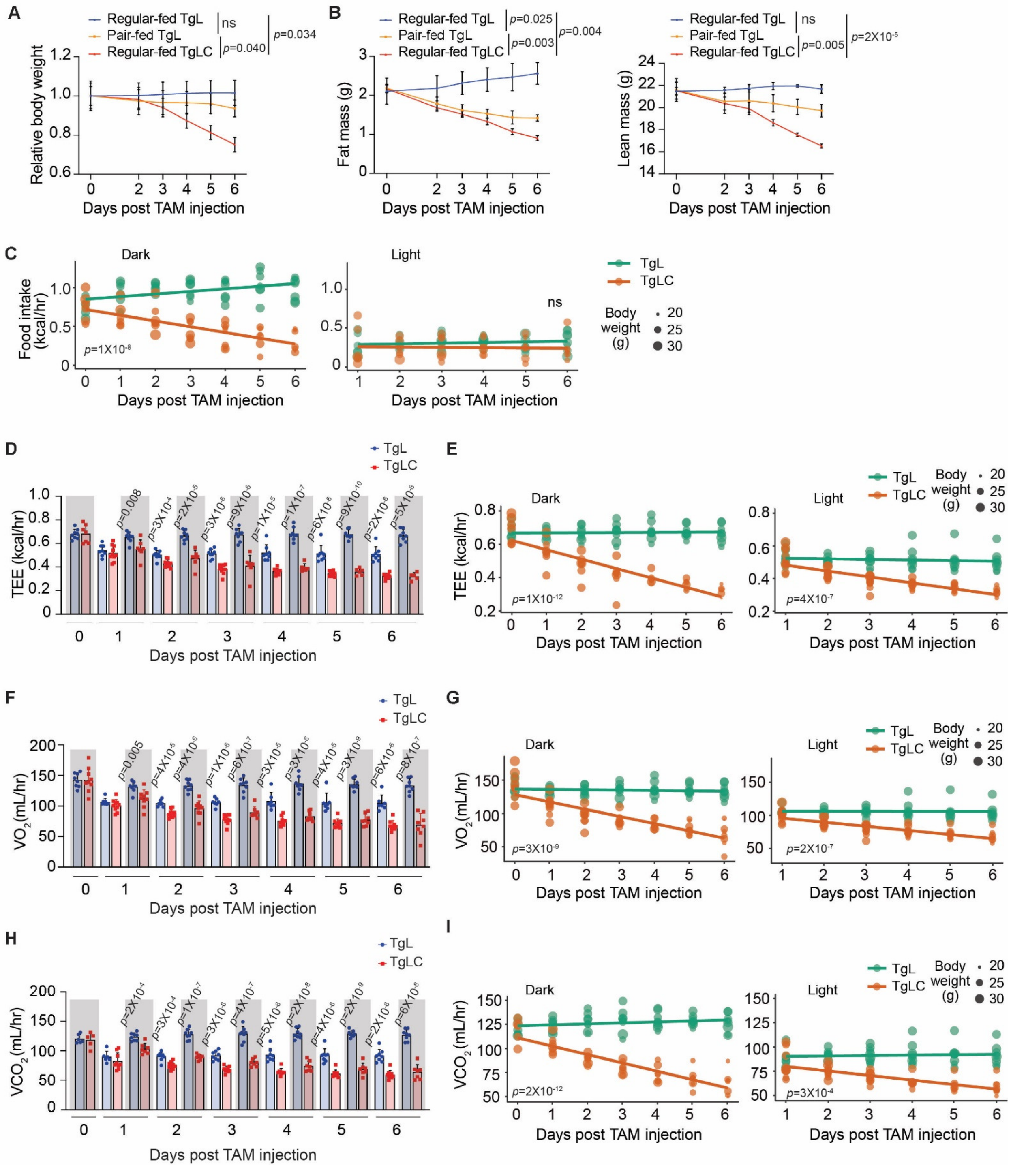

Supplementary Figure 2. Body composition change in pair-fed TgL mice with TAM injection and statistical analysis of indirect calorimetry data obtained from TgL and TgLC mice with TAM injection.

A. Mouse body weight post TAM injection in regular-fed TgL, TgLC and pair-fed TgL mice (n=6/group). **B.** The change of fat mass (left) and lean mass (right) post TAM injection in regular-fed TgL, TgLC and pair-fed TgL mice (n=6/group). **C.** Linear regression analysis of food intake between TgL and TgLC mice post TAM injection. **D.** TEE of TgL and TgLC mice post TAM injection calculated without body weight normalization (n≥4/group). **E.** Linear regression analysis of TEE between TgL and TgLC mice post TAM injection. **F.** VO_2 of TgL and TgLC mice post TAM injection (n≥4/group). Values are hourly means per mouse. **G.** Linear regression analysis of VO_2 between TgL and TgLC mice post TAM injection. **H.** VCO_2 of TgL and TgLC mice post TAM injection (n≥4/group). Values are hourly means per mouse. **I.** Linear regression analysis of VCO_2 of TgL and TgLC mice post TAM injection. Both dark (left) and light cycles (right) are shown. Data are presented as mean ± SEM for **A** & **B**, and as mean ± SD for **D**, **F** & **H**. ns: non-significant. Each dot represents an individual mouse. Both female and male mice were used. For **A** & **B**: Two-way ANOVA followed by Sidak's multiple comparison test, for **D**, **F** & **H**: Two-tailed Student's *t*-test; and for **C**, **E**, **G** & **I**: Linear regression analysis that is equivalent to ANCOVA analysis was used. Source data are provided as Source Data file.

Supplementary Figure 3

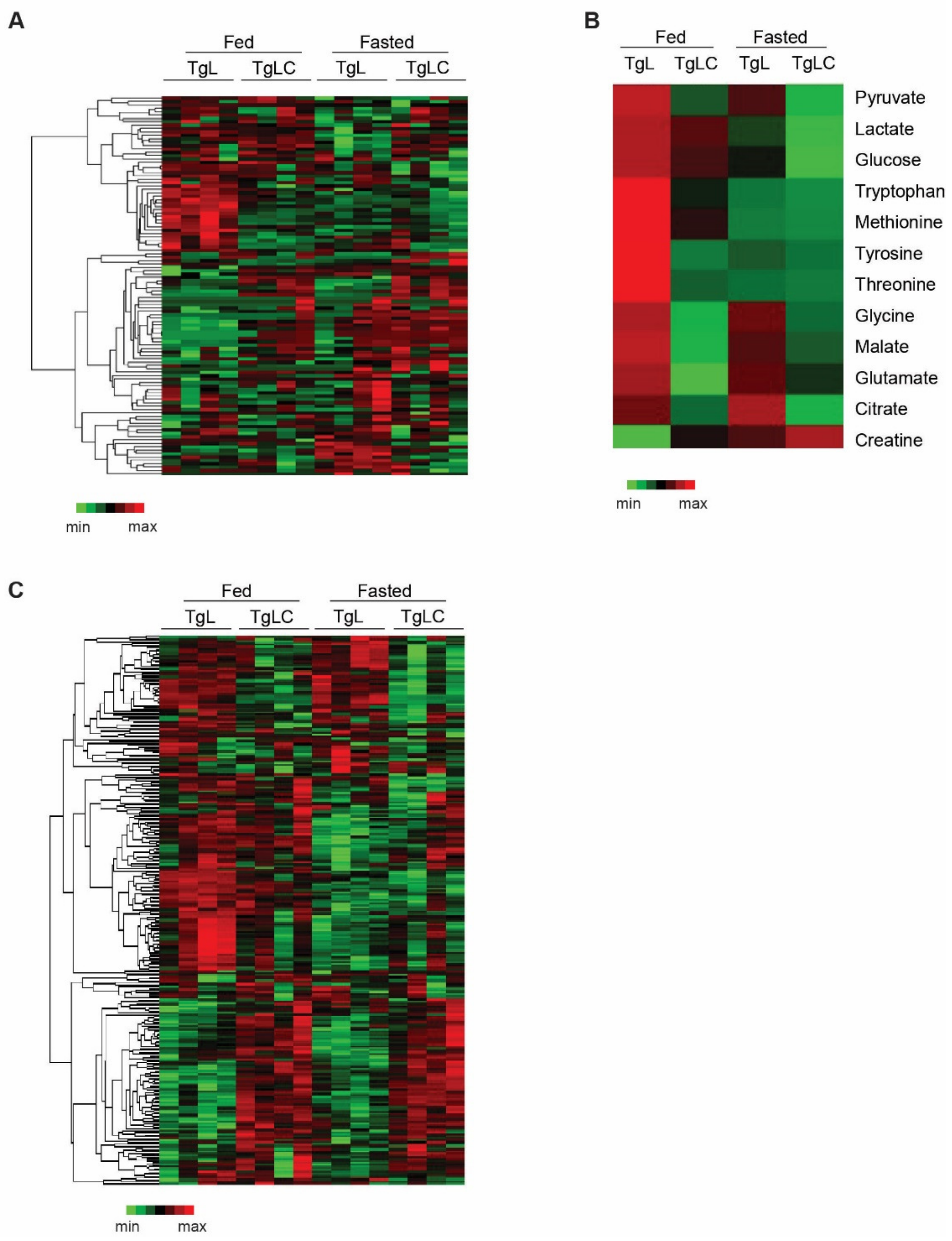

Supplementary Figure 3. LIF overexpression disrupts metabolic homeostasis in mice.

A. Heatmap of polar metabolic levels in the serum of TAM-injected TgL and TAM-injected TgLC mice under both fed and fasted conditions (n=4/group). **B.** Heatmap showing the levels of representative polar metabolites in the serum from TAM-injected TgL and TAM-injected TgLC mice under both fed and fasted conditions. Data are shown by the average of each group (n=4/group). **C.** Heatmap of lipid metabolic levels in the serum of TAM-injected TgL and TAM-

injected TgLC mice under both fed and fasted conditions (n=4/group). Both female and male mice were used.

Supplementary Figure 4

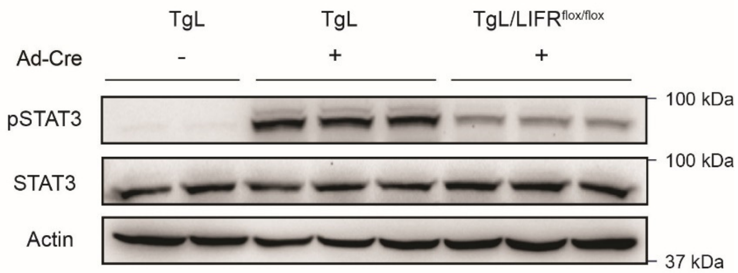

Supplementary Figure 4. The levels of pSTAT3 and total STAT3 protein in the liver of additional TgL and TgL/LIFR$^{flox/flox}$ mice with or without Ad-Cre injection determined by Western-blot assays. Both female and male mice were used. Uncropped Western-blot images are shown in **Supplementary Figure 9**.

Supplementary Figure 5

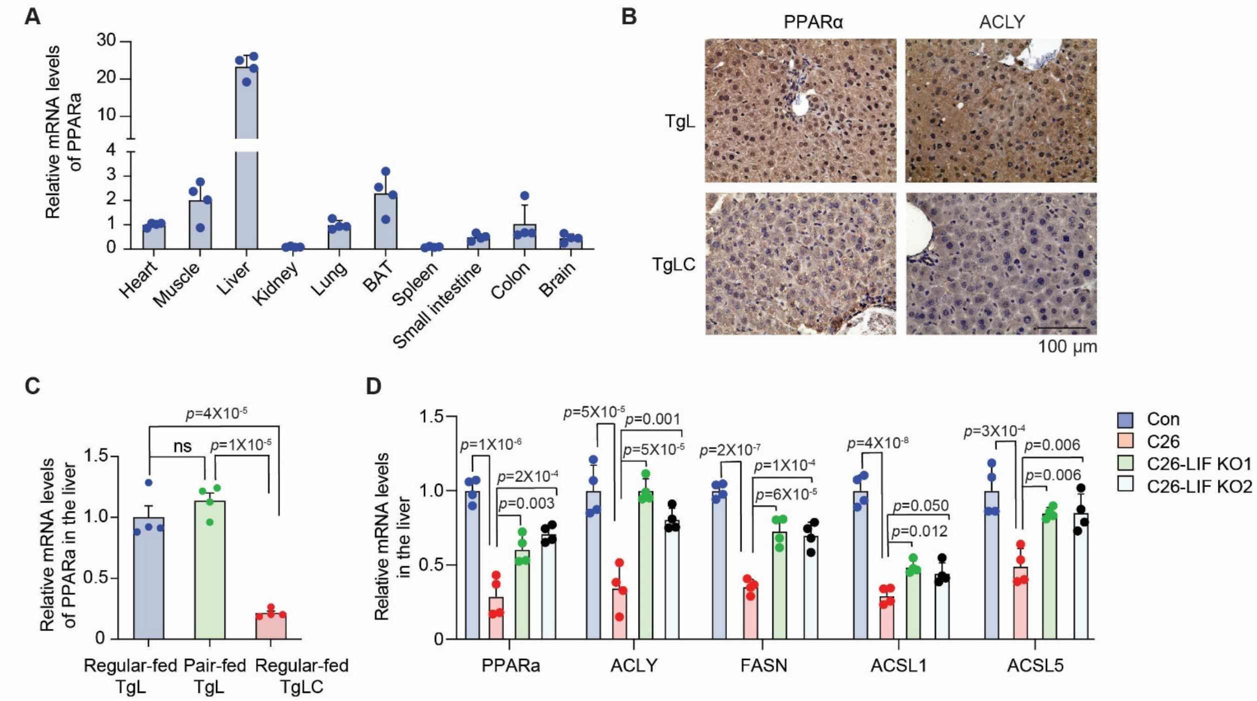

Supplementary Figure 5. **The mRNA levels of PPARa in different tissues, and the expression levels of PPARa and its target genes in the liver of TgL and TgLC mice with TAM injection and non-tumor bearing, C26, or C26-LIF KO tumor-bearing mice.**

A. PPARa mRNA levels in different tissues in C57BL/6 mice (n=4/group). **B.** PPARα and ACLY protein levels in TgL and TgLC mice with TAM injection determined by IHC staining assays. At least three independent biological replicates were performed. **C.** Relative mRNA levels of PPARa in the liver of regular-fed TgL, TgLC and pair-fed TgL mice with TAM injection (n=4/group). **D.** Relative mRNA levels of PPARa and its representative target genes in the liver of non-tumor bearing, C26, or C26LIF KO tumor-bearing Balb/c mice (n=4/group). The mRNA levels of genes were measured by qPCR and normalized to β-actin. All data are presented as mean ± SD. Each dot represents an individual mouse. Both female and male mice were used. ns: non-significant. *p* value was determined by one-way ANOVA, followed by *t*-test with Tukey's multiple comparison adjustment. Source data are provided as Source Data file.

Supplementary Figure 6

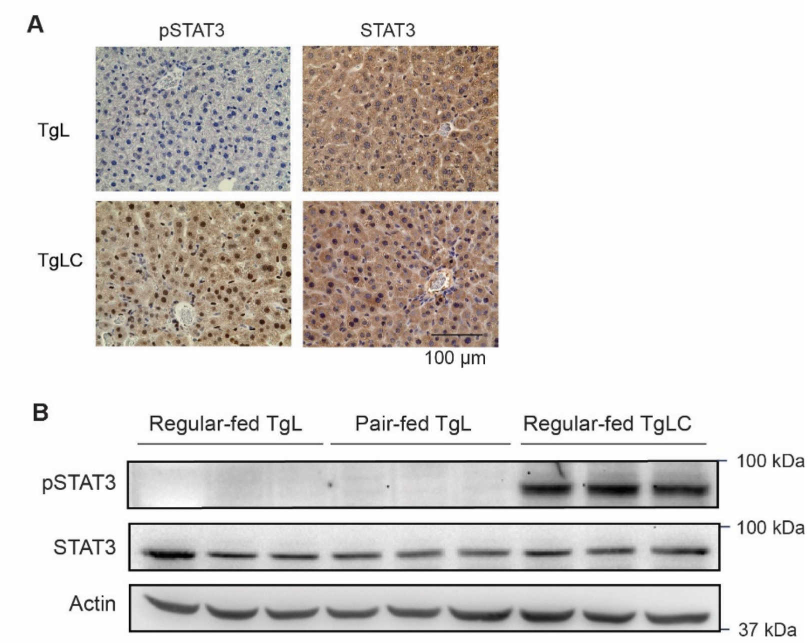

Supplementary Figure 6. **The STAT3 signaling in the liver of TgL and TgLC mice with TAM injection**.

A. The levels of pSTAT3 and total STAT3 protein in the liver of TgL and TgLC mice with TAM injection determined by IHC staining assays. **B.** The levels of pSTAT3 and total STAT3 protein in the liver of regular-fed TgL and TgLC mice and pair-fed TgL mice with TAM injection determined by Western-blot assays. Both female and male mice were used. At least three independent biological replicates were performed. Uncropped Western-blot images are shown in **Supplementary Figure 9**.

Supplementary Figure 7

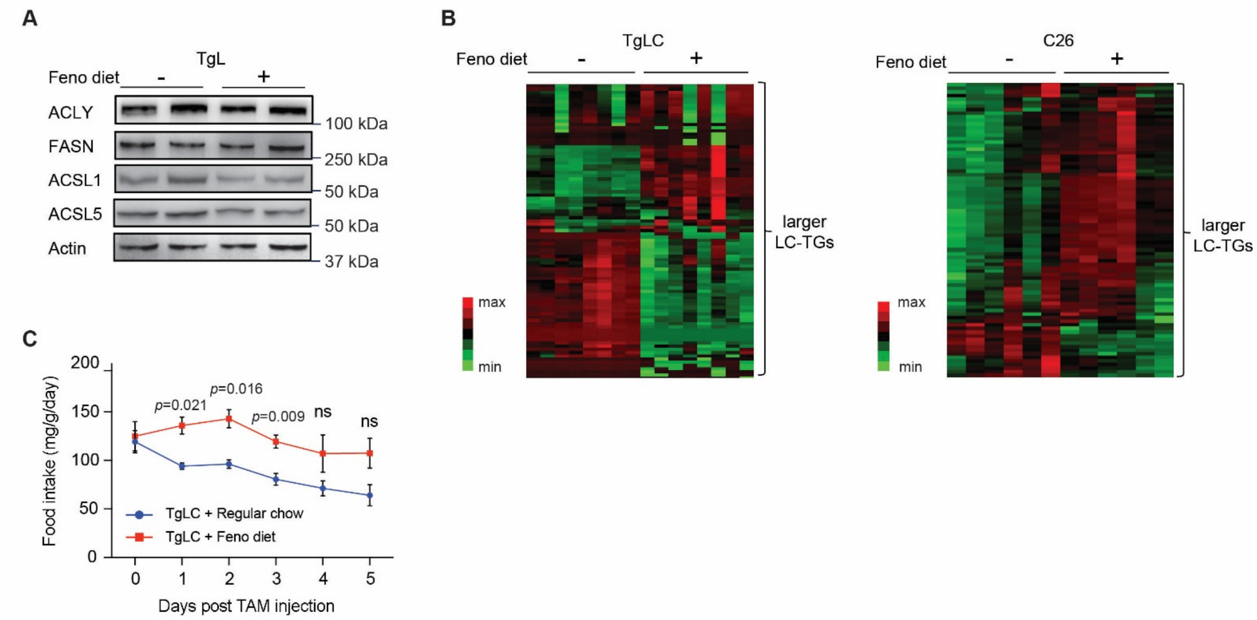

Supplementary Figure 7. **Fenofibrate restores lipid homeostasis in the liver of TAM-injected TgLC and C26 tumor-bearing mice.**

A. The protein levels of ACLY, FASN, ACSL1 and ACSL5 in the liver of TAM-injected TgL mice fed with regular chow or fenofibrate diet measured by Western-blot assays. At least three independent biological replicates were performed. **B.** Heatmap of TG levels in the liver of TAM-injected TgLC mice (n=8/group; left) and C26 tumor-bearing mice (n=6/group; right) fed with regular chow or fenofibrate diet. **C.** Food intake of TgLC mice fed with regular chow or fenofibrate diet post TAM injection (n=6/group). Data are presented as mean ± SEM. Both female and male mice were used. ns: non-significant. For **C**: Two-way ANOVA followed by Sidak's multiple comparison test. Uncropped Western-blot images are shown in **Supplementary Figure 9**. Source data are provided as Source Data file.

Supplementary Figure 8

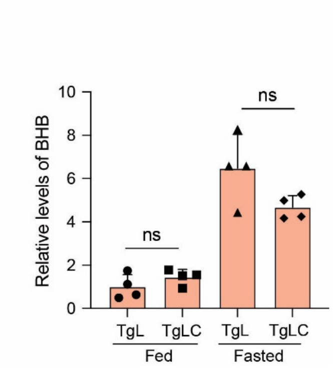

Supplementary Figure 8. The relative levels of BHB in the serum of TAM-injected TgL and TAM-injected TgLC mice (n=4/group). Two-tailed Student's *t*-test. Both female and male mice were used. ns: non-significant. Source data are provided as Source Data file.

Supplementary Figure 9

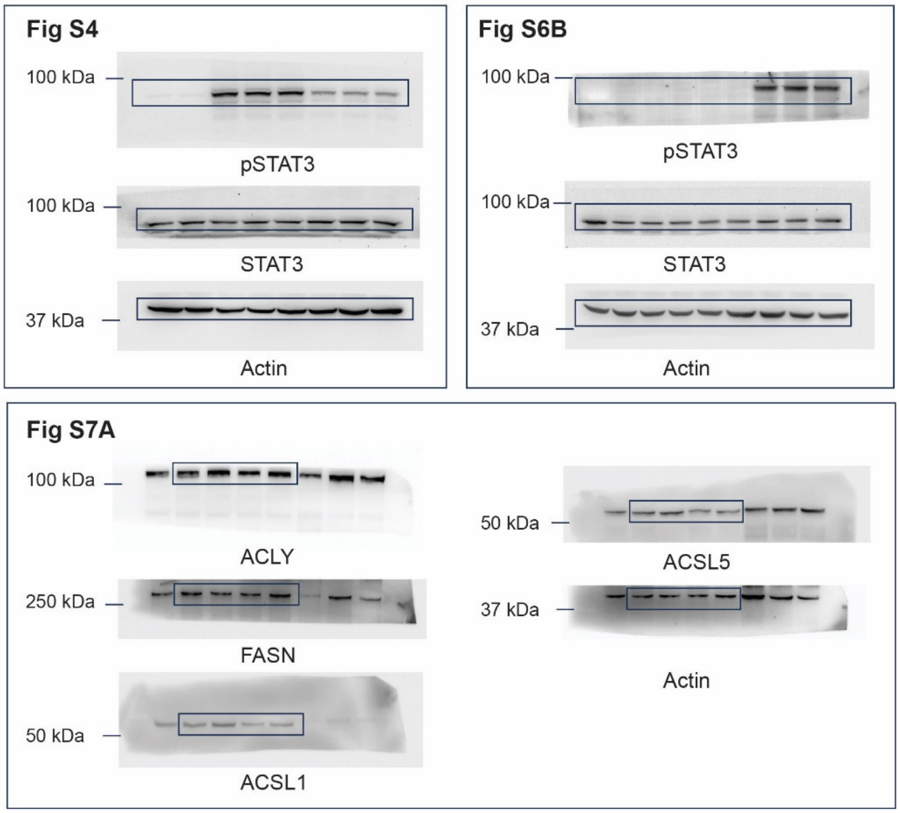

Supplementary Figure 9. Uncropped Western-blot images for panels in Supplementary Figures (Fig S4, Fig S6B, Fig S7A).

Supplementary Table 1. Composition of TGs

compound	mz	RT
* TG_NH4(44:0|13:0_15:0_16:0)	768.70789	15.953
* TG_NH4(44:1|12:0_16:0_16:1)	766.69037	15.753
* TG_NH4(44:2|10:0_16:0_18:2)	764.67535	15.569
* TG_NH4(45:0|14:0_15:0_16:0)	782.72296	16.076
* TG_NH4(45:1|14:0_15:0_16:1)	780.70715	15.878
* TG_NH4(46:0|14:0_16:0_16:0)	796.73816	16.238
* TG_NH4(46:1|14:0_16:0_16:1)	794.72113	16.014
* TG_NH4(46:2|14:0_14:1_18:1)	792.70605	15.817
* TG_NH4(46:3|10:0_18:1_18:2)	790.6908	15.643
* TG_NH4(47:1|15:0_16:0_16:1)	808.73914	16.142
* TG_NH4(47:2|15:0_15:1_17:1)	806.72272	15.946
* TG_NH4(48:0|14:0_16:0_18:0)	824.76923	16.559
* TG_NH4(48:1|14:0_16:0_18:1)	822.75391	16.3
* TG_NH4(48:2|14:0_16:0_18:2)	820.73767	16.076
* TG_NH4(48:3|14:0_16:1_18:2)	818.72321	15.884
* TG_NH4(48:4|12:0_18:2_18:2)	816.70728	15.698
* TG_NH4(49:1|15:0_16:0_18:1)	836.76862	16.458
* TG_NH4(49:2|15:0_16:1_18:1)	834.75183	16.253
* TG_NH4(49:3|15:0_16:1_18:2)	832.73718	16.011
* TG_NH4(50:0|16:0_16:0_18:0)	852.80115	16.892
* TG_NH4(50:1|16:0_16:0_18:1)	850.78503	16.599
* TG_NH4(50:2|16:0_16:1_18:1)	848.77051	16.356
* TG_NH4(50:3|16:0_16:1_18:2)	846.75433	16.128
* TG_NH4(50:4|14:0_18:2_18:2)	844.73682	15.923
* TG_NH4(50:5|14:0_18:2_18:3)	842.72296	15.78
* TG_NH4(51:1|16:0_17:0_18:1)	864.80139	16.763
* TG_NH4(51:2|16:0_17:1_18:1)	862.78485	16.512
* TG_NH4(51:3|15:0_18:1_18:2)	860.7691	16.261
* TG_NH4(51:4|15:0_18:2_18:2)	858.75507	16.049
* TG_NH4(52:0|16:0_18:0_18:0)	880.83185	17.26
* TG_NH4(52:1|16:0_18:0_18:1)	878.8175	16.938
* TG_NH4(52:2|16:0_18:1_18:1)	876.80084	16.648
* TG_NH4(52:3|16:0_18:1_18:2)	874.78503	16.406
* TG_NH4(52:4|16:0_18:2_18:2)	872.77026	16.182
* TG_NH4(52:5|16:0_18:2_18:3)	870.75354	15.995
* TG_NH4(52:6|16:1_18:2_18:3)	868.73724	15.847
* TG_NH4(52:7|18:1_16:3_18:3)	866.72186	15.705
* TG_NH4(53:1|17:0_18:0_18:1)	892.83246	17.128

* TG_NH4(53:2|16:0_18:1_19:1)	890.81775	16.819
* TG_NH4(53:3|17:0_18:1_18:2)	888.8009	16.553
* TG_NH4(53:4|17:1_18:1_18:2)	886.78442	16.316
* TG_NH4(53:5|18:1_17:2_18:2)	884.76886	16.103
* TG_NH4(54:0|16:0_18:0_20:0)	908.86499	17.67
* TG_NH4(54:1|16:0_20:0_18:1)	906.84839	17.323
* TG_NH4(54:2|16:0_18:1_20:1)	904.83167	17.001
* TG_NH4(54:3|18:0_18:1_18:2)	902.81494	16.714
* TG_NH4(54:4|18:1_18:1_18:2)	900.79865	16.464
* TG_NH4(54:5|18:1_18:2_18:2)	898.78583	16.232
* TG_NH4(54:6|16:0_18:2_20:4)	896.77032	16.135
* TG_NH4(54:6|18:1_18:2_18:3)	896.77051	16.051
* TG_NH4(54:7|18:2_18:2_18:3)	894.75238	15.941
* TG_NH4(54:8|16:1_18:2_20:5)	892.73743	15.777
TG_NH4(55:1|16:0_21:0_18:1)	920.86542	17.511
TG_NH4(55:2|18:0_19:0_18:2)	918.8479	17.169
TG_NH4(55:3|19:0_18:1_18:2)	916.83063	16.875
TG_NH4(55:7|16:0_18:2_21:5)	908.76862	16.083
TG_NH4(56:1|16:0_22:0_18:1)	934.87836	17.722
TG_NH4(56:10|18:2_18:3_20:5)	916.73822	15.697
TG_NH4(56:2|18:0_18:1_20:1)	932.86493	17.373
TG_NH4(56:3|18:0_20:1_18:2)	930.84851	17.047
TG_NH4(56:4|18:1_20:1_18:2)	928.83215	16.764
TG_NH4(56:5|18:0_18:1_20:4)	926.81464	16.629
TG_NH4(56:6|16:0_18:1_22:5)	924.80072	16.398
TG_NH4(56:7|18:1_18:2_20:4)	922.78351	16.251
TG_NH4(56:8|18:1_18:2_20:5)	920.76965	16.051
TG_NH4(56:9|16:1_18:2_22:6)	918.75232	15.835
TG_NH4(57:1|16:0_23:0_18:1)	948.89423	17.923
TG_NH4(58:1|16:0_24:0_18:1)	962.91235	18.171
TG_NH4(58:10|18:1_20:4_20:5)	944.77008	15.926
TG_NH4(58:11|18:2_20:4_20:5)	942.75238	15.774
TG_NH4(58:2|22:0_18:1_18:1)	960.89618	17.787
TG_NH4(58:3|22:0_18:1_18:2)	958.87964	17.436
TG_NH4(58:4|18:1_22:1_18:2)	956.86237	17.111
TG_NH4(58:6|18:0_18:2_22:4)	952.83331	16.67
TG_NH4(58:7|16:0_18:1_24:6)	950.81702	16.477
TG_NH4(58:8|18:1_18:1_22:6)	948.80048	16.312
TG_NH4(58:9|18:1_18:2_22:6)	946.78406	16.107
TG_NH4(60:10|18:1_20:4_22:5)	972.80115	16.245
TG_NH4(60:11|18:1_20:4_22:6)	970.78589	16.049

TG_NH4(60:12|18:2_20:4_22:6)	968.76953	15.878
TG_NH4(60:13|18:2_20:5_22:6)	966.75311	15.71
TG_NH4(60:8|18:1_20:1_22:6)	976.83142	16.555
TG_NH4(60:9|18:1_18:2_24:6)	974.81519	16.352
TG_NH4(62:13|18:1_22:6_22:6)	994.78516	15.983
TG_NH4(62:14|18:2_22:6_22:6)	992.76801	15.805
TG_NH4(64:16|20:4_22:6_22:6)	1016.76892	15.759
TG_NH4(66:18|22:6_22:6_22:6)	1040.77075	15.707

*: small LC-TGs

Supplementary Table 2. Sequences of primers and sgRNAs used in this study

Mouse genotyping primers	
mLIF-F	CAACTGGCACAGCTCAATGG
mLIF-R	ATGCGACCATCCGATACAGC

CRISPR sgRNAs	
sgRNA 1-F	CACCGGCATGGGTGGCGTATGGCAC
sgRNA 1-R	AAACGTGCCATACGCCACCCATGCC
sgRNA 2-F	CACCGGAACCAGATCAAGAATCAAC
sgRNA 2-R	AAACGTTGATTCTTGATCTGGTTCC

Chip assays primers	
Chip-F	TCCAAATGTCTGGTAGAACTGG
Chip-R	GAGAGGGTGCTTGGTTGTAG
Negative control F	GATGGAGCCCATTCCTGAACC
Negative control R	CCCTGTACTTATCCAGGCAGA

qPCR primers	
ACLY F	ACCCTTTCACTGGGGATCACA
ACLY R	GACAGGGATCAGGATTTCCTTG
FASN F	GGAGGTGGTGATAGCCGGTAT
FASN R	TGGGTAATCCATAGAGCCCAG
PPARa F	AGAGCCCCATCTGTCCTCTC
PPARa R	ACTGGTAGTCTGCAAAACCAAA
ACSL1 F	TGCCAGAGCTGATTGACATTC
ACSL1 R	GGCATACCAGAAGGTGGTGAG
ACSL5 F	TCCTGACGTTTGGAACGGC
ACSL5 R	CTCCCTCAATCCCCACAGAC
β-actin F	GAACCCTAAGGCCAACCGTGAAAAGAT
β-actin R	GCAGGATGGCGTGAGGGAGAGCA
