# Supplementary material for: Leukemia inhibitory factor suppresses hepatic de novo lipogenesis and induces cachexia in mice
Source: Nat Commun. 2024 Jan 20;15:627. doi: 10.1038/s41467-024-44924-w (PMC10799847; doi:10.1038/s41467-024-44924-w)
Supplement: Supplementary file 3 — Reporting Summary [file 41467_2024_44924_MOESM3_ESM.pdf]

nature portfolio

Corresponding author(s): Wenwei Hu and Zhaohui Feng

Last updated by author(s): 1/5/2024

Reporting Summary

Nature Portfolio wishes to improve the reproducibility of the work that we publish. This form provides structure for consistency and transparency in reporting. For further information on Nature Portfolio policies, see our Editorial Policies and the Editorial Policy Checklist.

Statistics

For all statistical analyses, confirm that the following items are present in the figure legend, table legend, main text, or Methods section.

n/a	Confirmed	
☐	☒	The exact sample size (*n*) for each experimental group/condition, given as a discrete number and unit of measurement
☐	☒	A statement on whether measurements were taken from distinct samples or whether the same sample was measured repeatedly
☐	☒	The statistical test(s) used AND whether they are one- or two-sided<br>*Only common tests should be described solely by name; describe more complex techniques in the Methods section.*
☒	☐	A description of all covariates tested
☐	☒	A description of any assumptions or corrections, such as tests of normality and adjustment for multiple comparisons
☐	☒	A full description of the statistical parameters including central tendency (e.g. means) or other basic estimates (e.g. regression coefficient) AND variation (e.g. standard deviation) or associated estimates of uncertainty (e.g. confidence intervals)
☐	☒	For null hypothesis testing, the test statistic (e.g. *F*, *t*, *r*) with confidence intervals, effect sizes, degrees of freedom and *P* value noted<br>*Give P values as exact values whenever suitable.*
☒	☐	For Bayesian analysis, information on the choice of priors and Markov chain Monte Carlo settings
☒	☐	For hierarchical and complex designs, identification of the appropriate level for tests and full reporting of outcomes
☒	☐	Estimates of effect sizes (e.g. Cohen's *d*, Pearson's *r*), indicating how they were calculated

Our web collection on statistics for biologists contains articles on many of the points above.

Software and code

Policy information about availability of computer code

Data collection	EchoMRI-100H
Data analysis	GraphPad Prism 9

For manuscripts utilizing custom algorithms or software that are central to the research but not yet described in published literature, software must be made available to editors and reviewers. We strongly encourage code deposition in a community repository (e.g. GitHub). See the Nature Portfolio guidelines for submitting code & software for further information.

Data

Policy information about availability of data

All manuscripts must include a data availability statement. This statement should provide the following information, where applicable:

- Accession codes, unique identifiers, or web links for publicly available datasets
- A description of any restrictions on data availability
- For clinical datasets or third party data, please ensure that the statement adheres to our policy

Source data are provided with this paper. RNA-seq data generated in this study have been deposited in the Gene Expression Omnibus (GEO) database under accession number GSE245198 (https://www.ncbi.nlm.nih.gov/geo/query/acc.cgi?acc=GSE245198). Metabolomics data have been deposited at the Metabolomics Workbench under Project ID: PR001766 (https://www.metabolomicsworkbench.org/data/DRCCMetadata.php?Mode=Project&ProjectID=PR001766).

Research involving human participants, their data, or biological material

Policy information about studies with human participants or human data. See also policy information about sex, gender (identity/presentation), and sexual orientation and race, ethnicity and racism.

Reporting on sex and gender	NA
Reporting on race, ethnicity, or other socially relevant groupings	NA
Population characteristics	NA
Recruitment	NA
Ethics oversight	NA

Note that full information on the approval of the study protocol must also be provided in the manuscript.

Field-specific reporting

Please select the one below that is the best fit for your research. If you are not sure, read the appropriate sections before making your selection.

☒ Life sciences ☐ Behavioural & social sciences ☐ Ecological, evolutionary & environmental sciences

For a reference copy of the document with all sections, see nature.com/documents/nr-reporting-summary-flat.pdf

Life sciences study design

All studies must disclose on these points even when the disclosure is negative.

Sample size	Sample sizes were chosen based on the power calculation.
Data exclusions	No data exclusions.
Replication	Experimental finding were replicated with at least 3 independent biological repeats.
Randomization	Animals were randomly assigned to different treatment groups.
Blinding	The investigators were blinded to the group allocation during experiments and when assessing outcomes.

Reporting for specific materials, systems and methods

We require information from authors about some types of materials, experimental systems and methods used in many studies. Here, indicate whether each material, system or method listed is relevant to your study. If you are not sure if a list item applies to your research, read the appropriate section before selecting a response.

Materials & experimental systems

n/a	Involved in the study	
☐	☒	Antibodies
☐	☒	Eukaryotic cell lines
☒	☐	Palaeontology and archaeology
☐	☒	Animals and other organisms
☒	☐	Clinical data
☒	☐	Dual use research of concern
☒	☐	Plants

Methods

n/a	Involved in the study	
☒	☐	ChIP-seq
☒	☐	Flow cytometry
☒	☐	MRI-based neuroimaging

Antibodies

Antibodies used	anti-PPARa (Abcam, Cata# ab24509), anti-ACLY (Santa Cruz, Cata# sc-517267), anti-FASN (Santa Cruz, Cata# sc-48357), anti-ACSL1 (cell signaling, Cata# 4047S), anti-ACSL5 (Santa Cruz, Cata# sc-365478), anti-PPARa (Thermo Fisher Scientific, Cata# MA5-37652), anti-STAT3 (Santa Cruz, Cata# sc-8019), anti-pSTAT3 (cell signaling, Cata# 9145S), anti-STAT1 (cell signaling, Cata# 14994S), anti-pSTAT1 (cell signaling, Cata# 9177S), anti-STAT4 (cell signaling, Cata# 2653S), anti-pSTAT4 (cell signaling, Cata# 4134S), anti-AKT

(Santa Cruz, Cata# sc-5298), anti-pAKT (cell signaling, Cata# 9018S), anti-pERK (cell signaling, Cata# 4376), anti-ERK (cell signaling, Cata# 9102), anti-pMAPK (cell signaling, Cata# 4511), anti-MAPK (cell signaling, Cata# 9212) and anti-β-actin (Sigma, Cata# A5441) antibodies

Validation

Validation of all commercial antibodies are available at the manufacturer's website. The reference for each antibody is listed below:
anti-PPARa (Abcam, Cata# ab24509)
https://www.abcam.com/products/primary-antibodies/ppar-alpha-antibody-ab24509.html
anti-ACLY (Santa Cruz, Cata# sc-517267)
https://www.scbt.com/p/atp-citrate-synthase-antibody-5f8d11
anti-FASN (Santa Cruz, Cata# sc-48357)
https://www.scbt.com/p/fatty-acid-synthase-antibody-g-11
anti-ACSL1 (cell signaling, Cata# 4047S)
https://www.cellsignal.com/products/primary-antibodies/acsl1-antibody/4047?_requestid=992441
anti-ACSL5 (Santa Cruz, Cata# sc-365478)
https://www.scbt.com/p/acsl5-antibody-a-2
anti-PPARa (Thermo Fisher Scientific, Cata# MA5-37652)
https://www.thermofisher.com/antibody/product/PPAR-alpha-Antibody-clone-1331CT894-186-143-Monoclonal/MA5-37652
anti-STAT3 (Santa Cruz, Cata# sc-8019)
https://www.scbt.com/p/stat3-antibody-f-2?gad_source=1&gclid=Cj0KCQjw1aOpBhCOARIsACXYv-fqQjEpKIvJ5hhm2gKwFfnhLGVlrD36zGAOoYOpdFDwFlpMN--8xyYaAkPWEALw_wcB
anti-pSTAT3 (cell signaling, Cata# 9145S)
https://www.cellsignal.com/products/primary-antibodies/phospho-stat3-tyr705-d3a7-xp-rabbit-mab/9145
anti-STAT1 (cell signaling, Cata# 14994S)
https://www.cellsignal.com/products/primary-antibodies/stat1-d1k9y-rabbit-mab/14994
anti-pSTAT1 (cell signaling, Cata# 9177S)
https://www.cellsignal.com/products/primary-antibodies/phospho-stat1-ser727-antibody/9177
anti-STAT4 (cell signaling, Cata# 2653S)
https://www.cellsignal.com/products/primary-antibodies/stat4-c46b10-rabbit-mab/2653
anti-pSTAT4 (cell signaling, Cata# 4134S)
https://www.cellsignal.com/products/primary-antibodies/phospho-stat4-tyr693-d2e4-rabbit-mab/4134
anti-AKT (Santa Cruz, Cata# sc-5298)
https://www.scbt.com/p/akt1-antibody-b-1
anti-pAKT (cell signaling, Cata# 9018S)
https://www.cellsignal.com/products/primary-antibodies/phospho-akt1-ser473-d7f10-xp-rabbit-mab/9018
anti-pERK (cell signaling, Cata# 4376)
https://www.cellsignal.com/products/primary-antibodies/phospho-p44-42-mapk-erk1-2-thr202-tyr204-20g11-rabbit-mab/4376
anti-ERK (cell signaling, Cata# 9102)
https://www.cellsignal.com/products/primary-antibodies/p44-42-mapk-erk1-2-antibody/9102
anti-pMAPK (cell signaling, Cata# 4511)
https://www.cellsignal.com/products/primary-antibodies/phospho-p38-mapk-thr180-tyr182-d3f9-xp-rabbit-mab/4511
anti-MAPK (cell signaling, Cata# 9212)
https://www.cellsignal.com/products/primary-antibodies/p38-mapk-antibody/9212?Ntk=Products&Ntt=9212
anti-β-actin (Sigma, Cata# A5441)
https://www.sigmaaldrich.com/US/en/product/sigma/a5441?gclid=Cj0KCQjw1aOpBhCOARIsACXYv-dAMtcOobDEgNNcr99Y71kEEKJwJ8u6GBeYBoyE43xYbfj_G5lTcv8aArJhEALw_wcB

Eukaryotic cell lines

Policy information about cell lines and Sex and Gender in Research

Cell line source(s)	C26 murine colon carcinoma cells (Cell lines service, Cata# 400156)
Authentication	C26 cells were confirmed for the high expression levels of LIF and its ability to induce cachexia in tumor-bearing mice.
Mycoplasma contamination	The cell line used in this study were tested negative for mycoplasma contamination.
Commonly misidentified lines (See ICLAC register)	No commonly misidentified cell lines were used in this study.

Animals and other research organisms

Policy information about studies involving animals; ARRIVE guidelines recommended for reporting animal research, and Sex and Gender in Research

Laboratory animals	Wild type C57BL6/J mice, Balb/c mice, and R26-CreERT2 mice (Stock No: 008463) were obtained from the Jackson Laboratory. LIFRflox/flox mice were obtained from The European Mouse Mutant Archive (EM: 09032). TgL mice were generated at Rutgers Transgenic Mouse Facility. Eight to ten-week-old mice were used for experiments.
Wild animals	No wild animal was involved in this study.
Reporting on sex	As cachexia occurs in both males and females, both males and female mice were used.
Field-collected samples	Field-collected samples were not involved in this study.
Ethics oversight	All mouse experiments were approved by the Institutional Animal Care and Use Committee (IACUC) of Rutgers University.

Note that full information on the approval of the study protocol must also be provided in the manuscript.
